# Toxicity and Sublethal Effects of *Piper hispidinervum* Essential Oil on Behavioral and Physiological Responses of *Sitophilus zeamais* Populations

**DOI:** 10.3390/molecules29174116

**Published:** 2024-08-30

**Authors:** Lucas M. Lopes, Adalberto H. de Sousa, Lêda R. A. Faroni, Marcus V. de A. Silva, Maria S. S. Ferraz, Vanderley B. dos Santos

**Affiliations:** 1 Center of Biological and Natural Sciences, Universidade Federal do Acre, Rio Branco 69920900, AC, Brazil; lucas.lopes@ufac.br (L.M.L.);; 2 Department of Agricultural Engineering, Universidade Federal de Viçosa, Viçosa 36570900, MG, Brazil; lfaroni@ufv.br (L.R.A.F.); suely.ferraz17@hotmail.com (M.S.S.F.)

**Keywords:** maize weevil, safrole, gas chromatography, chemical characterization, alternative control, biological activity, walking, respirometry

## Abstract

This study aimed to evaluate the toxicity of *Piper hispidinervum* essential oil (PHEO) against 11 Brazilian populations of *Sitophilus zeamais* (Coleoptera: Curculionidae). The effects of sublethal doses of PHEO on the behavior (walking and flying), respiration, and population growth (*r_i_*) of the insect populations were investigated. PHEO toxicity was determined through concentration–mortality bioassays, with mortality curves established using increasing PHEO concentrations ranging from 140.00 to 1000.00 μL kg^−1^. Behavior was evaluated based on walking distance, walking time, walking speed, walking time proportion, flight height, and flight takeoff success. Respiration was measured via the respiratory rate, while population growth (*r_i_*) was assessed through the instantaneous growth rate. All 11 populations of *S. zeamais* were susceptible to PHEO, showing no signs of resistance. The populations exhibited varying behavioral and physiological responses to sublethal exposure to PHEO, indicating different mitigation strategies. The results confirm that PHEO possesses insecticidal potential for controlling *S. zeamais* populations. However, the observed behavioral and physiological responses should be considered when establishing control measures in pest management programs for stored products.

## 1. Introduction

The maize weevil (*Sitophilus zeamais* [Motschulsky]) is one of the most destructive pests of stored grains worldwide [1,2]. Controlling this insect has primarily relied on the application of organophosphate insecticides, pyrethroids, and methyl bromide (which is currently banned), as well as phosphine (PH_3_) [3]. However, the continuous and indiscriminate use of these insecticides has led to the development of resistant populations [4]. Although effective, synthetic insecticides have undesirable effects on non-target organisms and raise concerns regarding human health and the environment [5,6].

Several control methods have been widely promoted as an alternative to synthetic insecticides, including modified atmospheres, inert powders, biological control, repellent extracts, and powders [7]. Another important alternative is botanical insecticides [8,9]. Over 100,000 secondary metabolites with insecticidal properties have been identified from approximately 200,000 plant species worldwide, including alkaloids, terpenoids, flavonoids, and quinones [10]. These compounds have multiple modes of action against insects, including acute toxicity, repellency, and inhibitory properties of growth, feeding, development, and reproduction mechanisms [11].

*Piper* is the largest genus of the Piperaceae family, comprising approximately 1,000 species. The essential oils of certain *Piper* species hold commercial value for the pharmaceutical and fragrance industries and exhibit insecticidal properties owing to their chemical profile that is rich in terpenes, propenylphenols, and alkaloids [12,13,14]. The species *Piper hispidinervum* C.DC., known as *pimenta-longa* in Brazil, is a shrub distributed throughout South America, with a notable occurrence in the state of Acre, Brazil [15].

*P. hispidinervum* essential oil is remarkably rich in safrole, especially when extracted from the leaves (80% to 98% of the total content) and young branches [16,17]. Safrole is a phenylpropanoid used in the synthesis of piperonyl butoxide (PBO), a synergistic agent that stabilizes and enhances the insecticidal action of natural pyrethroids [18,19]. Previous studies on the *P. hispidinervum* essential oil (PHEO) have demonstrated its insecticidal effects on several insect pests, such as *S. zeamais* [20,21], *Callosobruchus maculatus* (Fabricius) [22], *Tenebrio molitor* (L.) [23], and *Spodoptera frugiperda* (J. E. Smith) [16].

Most current studies have focused on analyzing the lethal effects of synthetic and natural insecticides. However, few have explored their sublethal effects on the locomotor behavior and physiology of insects, which can vary significantly depending on the toxic compound and the characteristics of the natural or controlled environment [24,25]. Although behavior is rarely the focus of toxicological studies, it is known that insects can alter their responses to the sensory perception of insecticides in ways that may compromise the effectiveness of these products [25,26]. Thus, understanding the sublethal effects is crucial in an integrated pest management program, and associating sublethal exposures to potential insecticides with the physiological characteristics of insects can provide deeper insights into their mechanisms of action [27,28].

The respiratory rate and body mass of the insects may also impact the energy required for the physiological processes involved in generating defense mechanisms against insecticides [29,30]. Considering that these characteristics influence toxicity, efforts to understand insecticide effects on the locomotor behavior and physiological responses of insect populations under sublethal exposures to *P. hispidinervum* essential oil are worthwhile.

This study evaluated the toxicity of *P. hispidinervum* essential oil to 11 Brazilian populations of *S. zeamais*. The research further examined the behavioral, physiological, and instantaneous population growth rate (*r_i_*) responses of weevil populations exposed to the sublethal effects of the oil.

## 2. Results

### 2.1. PHEO Composition

The chromatographic analysis revealed six components in the PHEO. Safrole was the principal constituent, accounting for 93.0% of the identified compounds, followed by bicyclogermacrene (2.05%), pentadecane (1.60%), spathulenol (1.46%), p-cymen-8-ol (1.20%), and (E)-caryophyllene (0.69%) (Table 1). The absolute concentration of safrole was 85.0%, as determined via GC-FID based on the retention time.

### 2.2. PHEO Toxicity

The toxicity of PHEO was determined using concentration–mortality curves (Table 2). There was slight variation in toxicity among the populations, with TR ranging from 1.00 to 1.91 at LC_50_ and LC_95_. The population from Cristalina (GO) had the lowest LC_50_ value (255.79 μL kg^−1^), and it was therefore adopted as the standard susceptibility population. The probit model was adequate for analyzing the concentration–mortality data, as low χ^2^ values and high *p* values were observed for each concentration–mortality curve (χ^2^ < 5.86, *p* > 0.05). However, the population curve slope varied from 3.60 to 6.85, which suggests some toxicological heterogeneity among these populations.

### 2.3. Sublethal Effects of PHEO on Respiratory Rate

**Figure 1:**
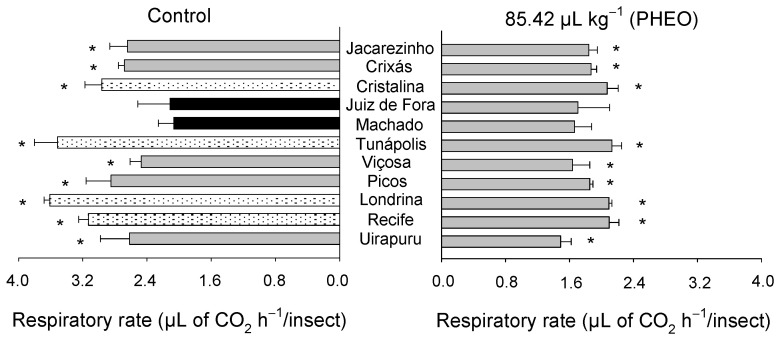
Respiratory rate of individuals of *Sitophilus zeamais* populations either unexposed (control) or exposed to a sublethal PHEO concentration (LC_30_ = 85.42 μL kg^−1^). Populations represented by mean bars of the same color did not differ according to the Scott–Knott test (*p* < 0.05). Asterisks indicate a significant difference between the populations exposed and unexposed to PHEO, according to the F-test (*p* < 0.05).

The respiratory rate (μL of CO_2_ h^−1^/insect) varied significantly among the *S. zeamais* populations (F_10;66_ = 5.31, *p* < 0.01), as well as between the PHEO-treated and the control groups (F_1;66_ = 106.17, *p* < 0.01). However, there was no significant interaction between the populations and the PHEO application (F_10;66_ = 1.34, *p* = 0.23) (Figure 1). Overall, the populations showed a reduction in respiratory rate when exposed to the essential oil, except for those from Juiz de Fora and Machado, which did not differ from the control.

**Figure 2:**
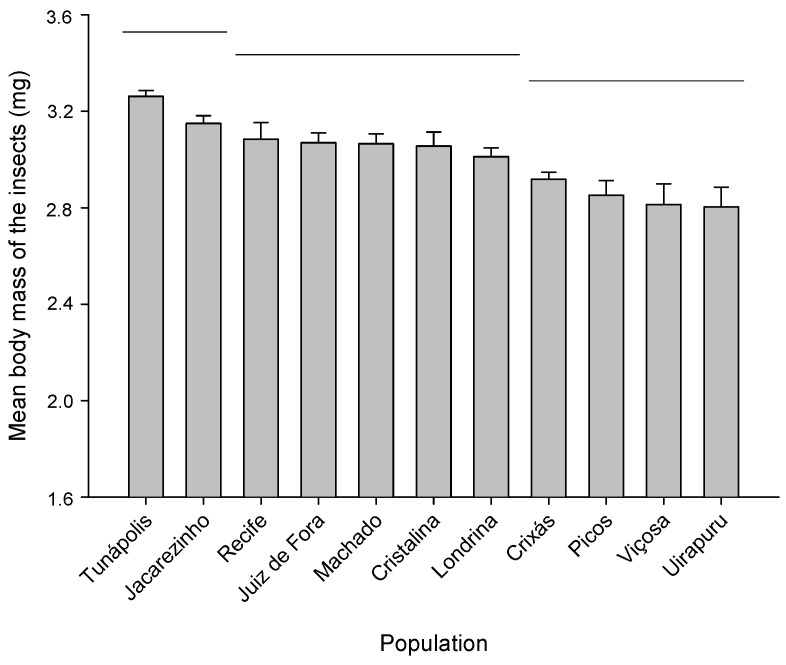
Mean body mass of *Sitophilus zeamais* insects from different populations. Means under the same horizontal bar did not differ, according to the Scott–Knott test (*p* < 0.05).

The individual body mass of the insects (mg) also differed significantly among the populations (F_10;77_ = 7.08, *p* < 0.01) (Figure 2), with those from Uirapuru, Viçosa, Picos, and Crixás showing lower body mass than the others.

### 2.4. Sublethal Effects of PHEO on the Instantaneous Population Growth Rate (r_i_)

**Figure 3:**
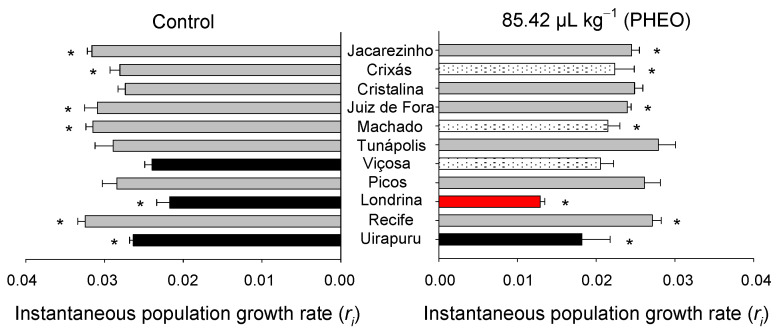
Instantaneous population growth rate (*r_i_*) of *Sitophilus zeamais* populations either unexposed (control) or exposed to a sublethal PHEO concentration (LC_30_ = 85.42 μL kg^−1^). Populations represented by mean bars of the same color did not differ according to the Scott–Knott test (*p* < 0.05). Asterisks indicate a significant difference between the population exposed and the unexposed to the PHEO according to the F-test (*p* < 0.05).

The *r_i_* varied significantly among the *S. zeamais* populations (F_10;66_ = 10.09, *p* < 0.01), as well as between the PHEO-treated and the control groups (F_1;66_ = 66.20, *p* < 0.01). However, no significant interaction was observed between the populations and the application of PHEO (F_10;66_ = 1.94, *p* = 0.10) (Figure 3). Overall, the populations showed a lower *r_i_* when exposed to PHEO, except for those from Viçosa, Cristalina, Picos, and Tunápolis, which did not differ from the control.

### 2.5. Sublethal Effects of PHEO on the Locomotor Behavior

**Figure 4:**
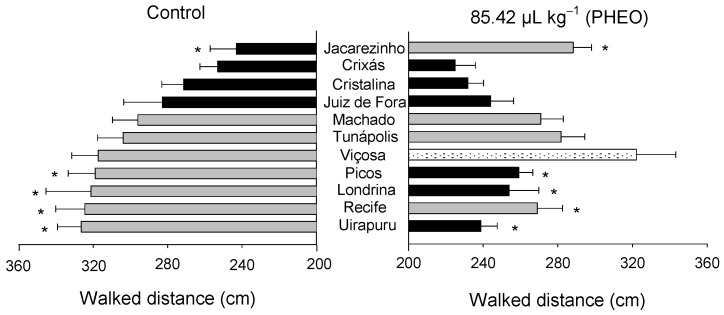
Walking distance by insects of *Sitophilus zeamais* populations either unexposed (control) or exposed to a sublethal PHEO concentration (LC_30_ = 85.42 μL kg^−1^). Populations represented by mean bars of the same color did not differ according to the Scott–Knott test (*p* < 0.05). Asterisks indicate a significant difference between the PHEO-exposed and the unexposed population according to the F-test (*p* < 0.05).

The walking distance by the insects varied significantly among the *S. zeamais* populations (F_10;418_ = 5.14, *p* < 0.01), as well as between the PHEO-treated and the control groups (F_1;418_ = 31.80, *p* < 0.01). A significant interaction was noticed between the populations and the PHEO treatment (F_10;418_ = 3.31, *p* < 0.01) (Figure 4). Response variations related to exposure to PHEO (or lack thereof) were observed among the populations. Those from Uirapuru, Londrina, Picos, and Viçosa covered shorter distances when exposed to PHEO. On the other hand, the distance traveled by the population from Jacarezinho treated with PHEO was greater than that of the untreated control group.

**Figure 5:**
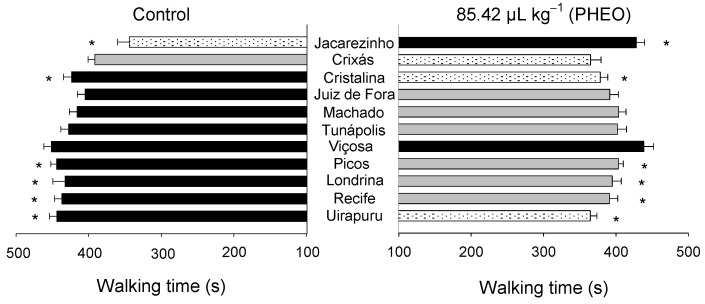
Walking time of *Sitophilus zeamais* populations either unexposed (control) or exposed to a sublethal PHEO concentration (LC_30_ = 85.42 μL kg^−1^). Populations represented by mean bars of the same color did not differ according to the Scott–Knott test (*p* < 0.05). Asterisks indicate a significant difference between the population exposed to PHEO and the unexposed according to the F-test (*p* < 0.05).

Walking time varied significantly among the *S. zeamais* populations (F1_0; 418_ = 4.93, *p* < 0.01), as well as between the PHEO-treated and control groups (F_1;418_ = 22.33, *p* < 0.01). A significant interaction was observed between the populations and the PHEO treatment (F_10; 418_ = 6.23, *p* < 0.01) (Figure 5). Walking time was shorter in the populations from Uirapuru, Recife, Londrina, Picos, and Cristalina exposed to PHEO. Conversely, the population from Jacarezinho showed a longer walking time when exposed to PHEO compared to the control.

**Figure 6:**
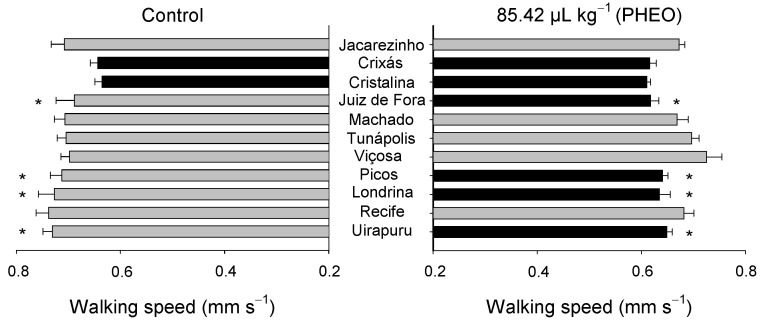
Walking speed of *Sitophilus zeamais* populations either unexposed (control) or exposed to a sublethal PHEO concentration (LC_30_ = 85.42 μL kg^−1^). Populations represented by mean bars of the same color did not differ according to the Scott-Knott test (*p* < 0.05). Asterisks indicate a significant difference between the population exposed to PHEO and that unexposed to the essential oil (*p* < 0.05).

Walking speed oscillated significantly among the *S. zeamais* populations (F_10;418_ = 4.52; *p* < 0.01), as well as between the PHEO-treated and the control groups (F_1;418_ = 26.10, *p* < 0.01). However, there was no significant interaction between the populations and the PHEO treatment (F_10;418_ = 1.57, *p* = 0.11) (Figure 6). Significant variations were observed in the populations depending on whether they were fully or partially exposed to PHEO. In the control groups, the populations from Cristalina and Crixás walked more slowly, but the results did not differ from the PHEO treatment. The populations from Juiz de Fora, Londrina, Picos, and Uirapuru exhibited less walking speed when exposed to PHEO, thus differing from the control.

**Figure 7:**
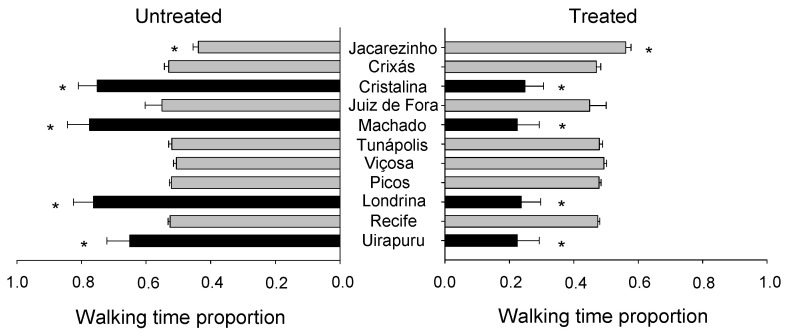
Walking time proportion of *Sitophilus zeamais* populations either untreated (control) or treated with a sublethal PHEO concentration (LC_30_ = 85.42 μL kg^−1^). Populations represented by mean bars of the same color did not differ according to the Scott–Knott test (*p* < 0.05). Asterisks indicate a significant difference between the PHEO-exposed and the unexposed populations according to the F-test (*p* < 0.05).

The walking time proportion did not vary significantly among the *S. zeamais* populations (F_10;418_ = 0.00; *p* = 1.00), but a significant variation was observed for the PHEO treatment (F_1;418_ = 106.20; *p* < 0.01). A significant interaction was found between these two factors (F_10;418_ = 15.56; *p* < 0.01) (Figure 7). Significant differences were noted among the populations partially exposed to PHEO (chance-choice test). The populations from Uirapuru, Machado, Londrina, and Cristalina walked proportionally shorter when exposed to PHEO, thus demonstrating a different behavioral strategy than the other populations that did not differ from the untreated group. On the contrary, the population from Jacarezinho walked for longer in the partially treated area when compared with the control.

### 2.6. Sublethal Effects of PHEO on Flight Height and Takeoff Success

**Figure 8:**
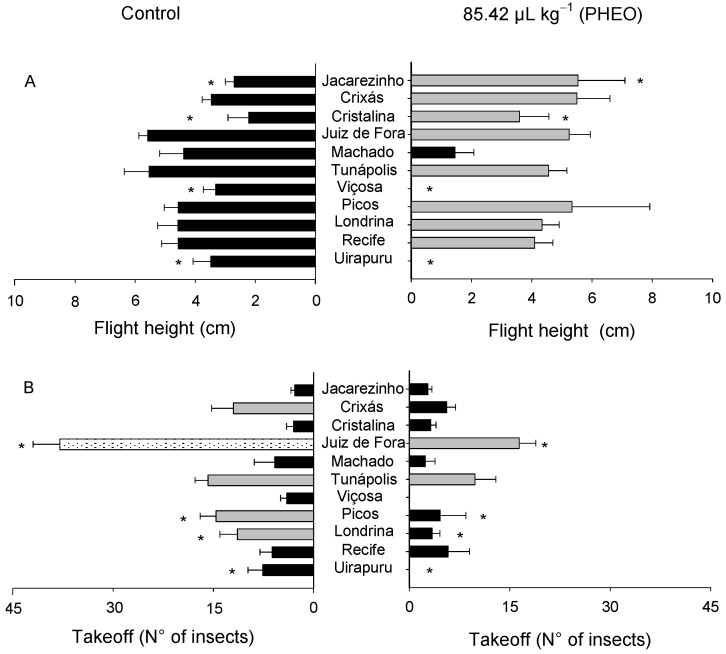
Flight height (**A**) and takeoff (number of insects that succeeded in flying) (**B**) of the *Sitophilus zeamais* populations untreated (control) and exposed to a sublethal PHEO concentration (LC_30_ = 85.42 μL kg^−1^). Populations represented by mean bars of the same color did not differ according to the Scott–Knott test (*p* < 0.05). Asterisks indicate a significant difference between the PHAO-treated and the untreated populations according to the F-test (*p* < 0.05).

The flight height and takeoff success varied significantly among the populations of *S. zeamais* (F_10; 88_ = 4.52, *p* < 0.01; F_10;88_ = 15.94, *p* < 0.01), as well as between the PHEO-treated and control groups (F_10;88_ = 1.33, *p* < 0.01; F_10;88_ = 31.51, *p* < 0.01). Significant interactions were observed between the populations and the PHEO treatment (F_10; 88_ = 3.01, *p* < 0.01; F_10;88_ = 2.94, *p* < 0.01, regarding flight height and takeoff success, respectively) (Figure 8A,B).

Significant variations were observed in the flight activity of the populations of *S. zeamais* exposed to PHEO. As for flight height, different avoidance strategies were adopted by the insects. The populations from Uirapuru, Viçosa, and Machado had the lowest averages for this variable, although the one from Machado did not differ from the control. On the other hand, the populations from Jacarezinho and Cristalina showed higher flight heights, differing significantly from the control.

In relation to takeoff (number of insects that succeed in flying), the populations from Uirapuru, Londrina, Picos, and Juiz de Fora displayed a lower takeoff activity when compared with the control. The other populations did not differ regarding takeoff, whether they were exposed to PHEO or not.

## 3. Discussion

Safrole accounted for 93.0% of the PHEO composition, followed by bicyclogermacrene (2.05%), pentadecane (1.60%), spatulenol (1.46%), p-cimen-8-ol (1.20%), and (E)-caryophyllene (0.69%). Safrole has already been reported as the major component of PHEO in other studies [14,32]. The insecticidal property of this substance has proved effective against insects that infest stored grains. Recent studies have indicated that safrole acts mainly as a neurotoxin, interfering with the nervous system of insects, eventually killing them [33]. In laboratory experiments, safrole caused disorientation, paralysis, and death to insects, including *S*. *zeamais* and *C. maculatus*, common pests in stored grains [34]. In addition to its effectiveness, safrole also has the advantage of being a natural and less toxic alternative to synthetic insecticides, thus contributing to food safety and reducing chemical residues in stored products [35].

All the 11 evaluated populations of *S*. *zeamais* were susceptible to PHEO, and their responses were relatively homogeneous, with low toxicity ratios for LC_50_ and LC_95_ (TR ≤ 1.91 times). These results indicate a uniform response to the bioinsecticide and rule out any evidence of cross-resistance with commercial pesticides, a phenomenon already reported in Brazilian populations of *S*. *zeamais* [36,37,38,39,40]. The absence of resistance to PHEO has also been verified in five populations of *S*. *zeamais* (LC_50_ < 7.36 µL L^−1^ and TR ≤ 1.6 times) [17]. The concentration–mortality curve slopes varied among the populations (3.60 to 6.85), showing some evidence of toxicological heterogeneity among the populations investigated. PHEO is not commonly used as a contact insecticide for controlling grain pests. Therefore, without selective pressure and cross-resistance with other insecticides, the toxicity response to PHEO in the evaluated populations seemed favorable for insect-pest management. The toxicity of PHEO applied via contact and fumigation to stored grain insect pests (*S*. *zeamais* and *T*. *molitor*) has already been reported in the literature [23,41].

Regarding insect body mass, significant differences were found among the 11 populations, with a variation of 16%. High body mass and respiration rate are indicators of substantial energy reserves and rapid mobilization of energy to sustain potentially conflicting physiological compensations, such as biochemical defense and reproduction mechanisms [3,41,42]. The reduction in the instantaneous growth rate (*r_i_*) of populations exposed to sublethal concentrations of PHEO (except for those from Viçosa, Cristalina, Picos, and Tunápolis) reinforces the idea that the allocation of reproductive energy to defense mechanisms against insecticides can impair the reproductive performance of these individuals [3,43,44,45]. Body mass has been identified as a trait influencing *r_i_* in populations of *S*. *zeamais* and, consequently, has a strong relationship with mitigating adaptive costs in some insecticide-resistant populations [24,25,45].

The behavioral patterns (walking and flight takeoff) of *S*. *zeamais* populations were influenced by sublethal exposure to PHEO. Researchers found different behavioral patterns when investigating the sublethal effects of *P. hispidinervum* essential oil on the behavior (walking and flying) of five populations of *S. zeamais* [17]. Alterations in the behavioral pattern may occur due to the action of some toxic compounds in essential oils or insecticides on the nervous system of insects, which may stimulate or reduce their mobility and, consequently, alter additional abilities to adapt to environments containing toxic compounds [24,25,26,46]. Behavioral responses to sublethal exposure to PHEO were population-dependent, revealing different strategies for mitigating the effects of essential oil exposure.

The walking patterns (walking distance, walking time, walking speed, and walking time proportion) of the populations from Uirapuru and Londrina were reduced in the presence of PHEO. Conversely, the population from Jacarezinho was more active regarding flight (flight height) and walking (walking distance, walking time, and walking time proportion). Braga et al. [30], when investigating the behavioral resistance of 11 Brazilian populations of *S*. *zeamais* to fenitrothion, found lower locomotor behavior levels and higher flight activity rates in the insects. These characteristics potentially minimize exposure to insecticides, especially within the same insect population, and lead to the emergence of behavioral resistance [47,48].

This behavioral resistance or behavioral escape can depend or not on stimuli [27,49]. Irritability and repellency are two types of behavioral resistance that depend on an insecticide stimulus (i.e., they require sensory stimulation) to elicit a flight response upon detecting a toxic substance, either with or without contact with it [47]. In the behavioral bioassay in arenas partially treated with PHEO, the populations from Uirapuru, Machado, Londrina, and Cristalina remained longer in the area not treated with the essential oil, thus confirming the repellent effect of PHEO on these populations. Plata-Rueda et al. [46], investigating the effects of the terpenoid constituents in cinnamon and clove essential oils, identified repellency effects on adults of *S. granarius* (L.). The detection of the insecticide in the environment may be associated with the learning capacity of the insects or genetic variation in peripheral receptors and central processing systems [27,50].

PHEO is a promising alternative bioinsecticide with a low potential for resistance development in the short term owing to the high susceptibility and low variability of response to this product. The different behavioral and physiological responses of the populations exposed to sublethal doses of PHEO indicate the existence of strategies that mitigate the effects of exposure to the essential oil. Therefore, instead of simply replacing commercial insecticides with essential oils, pest resistance management programs need to consider the behavioral and physiological responses of insect populations, as well as other biological aspects, to achieve satisfactory control levels.

## 4. Materials and Methods

### 4.1. Insect Populations

Eleven Brazilian populations of *S*. *zeamais* were collected in the municipalities of Cristalina, in the state of Goiás (GO); Crixás (GO); Jacarezinho, Paraná (PR); Juiz de For, Minas Gerais (MG); Londrina (PR); Machado (MG); Picos, Piauí (PI); Recife, Pernambuco (PE); Tunápolis, Santa Catarina (SC); Uirapuru (GO); and Viçosa (MG). The insects were reared in 1.5-liter glass flasks under constant temperature (27 °C ± 2 °C), relative humidity (70% ± 5%), and scotophase (24 h). Maize grains with a moisture content of 13%, on a wet basis (wb), were used as the food substrate. They were previously fumigated with phosphine (PH_3_) (Phostek, Bequisa, São Vicente, SP, Brazil) and kept at −18 °C to avoid reinfestation.

### 4.2. Obtaining and Extracting P. hispidinervum Essential Oil

*Piper hispidinervum* samples were collected in the rural area of the municipality of Bujari, Acre (AC), Brazil (9°42′17.26″ S latitude and 68°3′15.63″ W longitude). Bujari is 196 m above sea level and 23 km northwest of Rio Branco, the capital and largest city of the state. The plant material was harvested during the morning, in June 2017. A specimen was deposited at the UFACPZ Herbarium of the Federal University of Acre, in Rio Branco, under the registration number UFACPZ 20.647. The material was identified by Dr. Elsie Franklin Guimarães, who works at the Herbarium of the Botanical Garden of Rio de Janeiro (*Herbário RB*).

All botanical material was collected with the consent of the Brazilian Ministry of Environment and Climate Change (MMA), abiding by the Biodiversity Law No. 13,123/2015 and the Decree No. 8772/2016. The former addresses genetic heritage, protection and diligent access to local traditional knowledge, and conservation and sustainable use of biodiversity. Decree No. 88,772/2016 regulates the Biodiversity Law and created the National System for the Management of Genetic Heritage and Associated Traditional Knowledge (SisGen electronic system). It is worth noting that Dr. Adalberto Hipólito de Sousa obtained permission from SisGen (No. ADEFA9D) to collect botanical material from *P*. *hispidinervum*. The experiment with wild *P*. *hispidinervum*, including the collection of plant material, complies with the relevant institutional, national, and international guidelines and legislation.

*Piper hispidinervum* essential oil (PHEO) was extracted by hydrodistillation using a Clevenger-type apparatus coupled to a 5-liter volumetric flask and a heating mantle (0321a28, Quimis, Diadema, SP, Brazil). The PHEO was separated from the emulsion via decantation in a separation funnel, and anhydrous sodium sulfate was used for the analysis (Synth, Diadema, SP, Brazil). The essential oil obtained was stored inside an amber flask at 4 °C ± 1 °C.

### 4.3. PHEO Composition

The PHEO was analyzed using gas chromatography combined with mass spectrometry (GC-MS) using the QP2010 system (Shimadzu, Kyoto, Japan). The chromatographic conditions included using a fused-silica capillary column (30 m in length, 0.25 mm internal diameter) with an RTX^®^-5MS stationary phase (0.25 µm film thickness) and helium as the carrier gas applied at a flow rate of 1.2 mL min^−1^. The temperature at the injector was 220 °C, and the column was initially at 60 °C. The heating rate was set to increase by 2 °C min^−1^ up to 200 °C, and then by 5 °C min^−1^ up to 250 °C, remaining in this condition for 1 min. The mass spectra were obtained via electron impact at 70 eV, with range coverage from 29 to 400 (*m*/*z*). The chromatograph operated in full-scan mode, with a split ratio of 1:20. The total analysis time was 81 min.

The compounds were identified by comparing the resulting mass spectra with those listed in the NIST library and by visual interpretation [31]. They were confirmed using the Kovats Index (KI) and by comparison with the literature data (49451-U, Supelco, Bellefonte, PA, USA). The KI value of each compound was calculated based on the retention time of the compound and on the alkanes of the standard alkane solution. The relative percentage of each compound corresponded to the ratio between the area of each peak and the total area of all sample components.

### 4.4. Absolute Safrole Quantification

Quantifying absolute safrole in the PHEO employed a gas chromatograph with a flame ionization detector (GC/FID) (GC2014, Shimadzu, Tokyo, Japan). An analytical-grade safrole solution (Supelco, Darmstadt, Germany) was diluted in methanol to concoct solutions at five concentrations (0.25, 0.50, 1.00, 1.50, and 2.00 mg mL^−1^), which were analyzed in three replicates. PHEO diluted to 1 mg mL^−1^ in methanol (Vetec, UV/HPLC 99.9%, Verden, Germany) was also injected into the chromatograph in triplicate. The chromatographic conditions for safrole quantification were the following: 30 m long capillary column (DB-5, Shimadzu, Japan) with 0.25 mL internal diameter and 0.10 μm film thickness, nitrogen at 99.999% purity as the carrier gas (Air Products, São Paulo, Brazil) at a flow rate of 1.82 mL min^−1^, injector at 220 °C, and flame ionization detector at 300 °C with a split ratio of 1:5. The initial temperature of the column was 60 °C, and it was increased at a rate of 5 °C min^−1^ until 120 °Cl; it was kept at this condition for 1 min. The total analysis time was 12 min.

### 4.5. PHEO Toxicity Bioassays

The toxicity of PHEO to the 11 populations of *S. zeamais* was determined through concentration–mortality bioassays. Mortality curves were established using increasing PHEO concentrations (140, 250, 370, 490, 600, 800, and 1000 μL kg^−1^), as determined in preliminary tests.

A volume of 400 μL of PHEO was applied to 200 g of maize grains using a double-action gravity feed airbrush with an internal mixing system (model BC 60, Steula, São Paulo, Brazil) operating at 15 psi. The experimental units comprised glass containers (2.1 cm diameter × 7.4 cm height), each holding 20 g of maize. Twenty unsexed adult insects aged 1–3 weeks were placed into each glass flask and kept under constant conditions of temperature (27 °C ± 2 °C), relative humidity (70% ± 5%), and scotophase (24 h). Six replicates were performed for each concentration, and the adult mortality was assessed 24 h after the beginning of the bioassays. Acetone was used in the control treatment.

### 4.6. Sublethal Effect of PHEO on Respirometry

The effect of sublethal exposure to PHEO (LC_30_ = 85.42 μL kg^−1^, based on the standard susceptibility population) on the respiratory rate was determined in the 11 populations of *S. zeamais*. The previous toxicity bioassay determined the standard susceptibility population (the one from Cristalina, GO). Initially, the insects were exposed to a sublethal PHEO concentration for 30 min in Petri dishes (9 cm × 2 cm) and subsequently transferred to respirometric chambers.

Carbon dioxide (CO_2_) production (μL of CO_2_ h^−1^/insect) was measured using a CO_2_ analyzer TR3C respirometer (Sable System International, Las Vegas, NV, USA), following a methodology adapted from previous studies [3,41]. This evaluation employed 25 mL respirometric chambers connected in a completely closed system, each containing 10 unsexed adult insects.

CO_2_ production was assessed after insects had been acclimatizing for 2 h in the chambers at 27 °C ± 2 °C. CO_2_-free air at a flow rate of 100 mL min^−1^ was circulated through each chamber for 2 min to remove all the CO_2_ produced inside each chamber. This air current forced all the CO_2_ molecules to pass through an infrared reader attached to the system, which continuously measured the CO_2_ produced by the insects and accumulated inside each chamber. The control comprised respirometric chambers with insects exposed to acetone, and it was used to normalize the respiratory rate data of each population.

After the CO_2_ measurement, the insects were removed from the chambers and weighed on an analytical balance (Sartorius BP 210D, Göttingen, Germany). The respiratory rate values were not normalized by body mass because this procedure masks the individual effect of the variables [51]. Four replicates were used for each population.

### 4.7. Sublethal Effect of PHEO on the Instantaneous Population Growth Rate (r_i_)

The effect of sublethal exposure to PHEO (LC_30_ = 85.42 μL kg^−1^) on the instantaneous population growth rate (*r_i_*) was determined in 11 populations of *S. zeamais*. The bioassays were performed in 0.8-liter glass flasks, each containing 250 g of maize. The grains had a moisture content of 13% (wb) and were previously treated with 85.42 μL kg^−1^ of PHEO or acetone (control). PHEO and acetone were sprayed onto the grain mass using a double-action gravity feed airbrush with an internal mixing system (model BC 60, Steula, São Paulo, Brazil). The working pressure used for spraying was 15 psi, and the volume of solution sprayed was 400 μL for each 200 g of maize.

After treatment, the grains were infested with 50 unsexed adult insects aged 1 to 3 weeks. Afterward, the flasks were stored in biochemical oxygen demand climatic chambers under constant conditions of temperature (27 °C ± 2 °C), relative humidity (70% ± 5%), and scotophase (24 h). Four replicates were performed for each treatment. In these bioassays, no insect was removed from the flasks, and the adult progenies were counted after 60 days of storage.

The instantaneous growth rate (*r_i_*) was calculated using the equation proposed by Walthall and Stark [52] (Equation (1)), considering the total number of insects obtained by the end of storage (60 days) and the initial number of insects in each population.

(1) $$r_{i}=\frac{\left[\ln\left(\frac{N_{f}}{N_{0}}\right)\right]}{\Delta T}$$

where: *N_f_* = final number of insects, *N*_0_ = initial number of insects, and *T* = time variation (number of evaluation days).

### 4.8. Sublethal Effect of PHEO on the Locomotor Behavior

The walking bioassays were performed in a room with artificial lighting and controlled temperature (27 °C ± 2 °C) for 8 to 17 h, following a methodology adapted from previous studies [10,46]. Two behavioral walking bioassays were performed in fully or partially (half) PHEO-treated arenas. The walking characteristics of each population were observed for 10 min in Petri dish arenas (9 cm in diameter and 2 cm in height). A filter paper (Quanty JP42-Blue Stripe, Stripe, San Francisco, CA, USA) (9 cm in diameter) was placed inside the culture dish, and its inner walls were coated with Teflon^®^ PTFE (DuPont, Wilmington, DE, USA) to prevent the insects from escaping.

In the fully treated arena bioassays, the filter paper was moistened with 1 mL of PHEO solution (LC_30_ = 85.42 μL kg^−1^), while the control was moistened with 1 mL of acetone. For the bioassay in a partially treated arena, the filter paper was initially moistened with 1 mL of acetone. Then, half of a filter paper disk was moistened with 1 mL of PHEO solution (LC_30_ = 85.42 μL kg^−1^) and glued over the first filter paper with a common glue stick.

The movement of each insect in the fully and partially treated arenas was monitored using a tracking system comprising a charge-coupled device camera, which recorded and transferred the images digitally to a computer (ViewPoint Life Sciences Inc., Montreal, QC, Canada). A total of 20 replicates were performed for each of the different treatments and populations, and each replicate consisted of an unsexed *S. zeamais* adult insect aged 1 to 3 weeks. The insects were placed individually at the center of the arena 2 min before starting the trial for their spatial recognition. The characteristics of distance traveled, walking time, and walking speed were evaluated in the fully treated arenas, while the walking time was assessed in the partially treated ones.

### 4.9. Sublethal Effect of PHEO on Flight Takeoff

The study employed a methodology adapted from previous studies [47,53]. The application of LC_30_ = 85.42 μL kg^−1^ of PHEO was performed in Petri dishes (9 cm in diameter and 2 cm in height). The culture dish was lined with a filter paper (Quanty JP42-Blue Stripe) (9 cm in diameter), and its inner walls were coated with Teflon^®^ PTFE (DuPont, Wilmington, DE, USA) to prevent the insects from escaping. The insects were released into the Petri dishes. They were placed at the bottom of chambers (13 cm wide × 20 cm high), which also had their inner walls coated with Teflon^®^ PTFE (DuPont, São Paulo, Brazil) or entomological glue (Bio Controle, São Paulo, Brazil) up to 2 cm to prevent the insects from climbing on them. Twenty replicates were performed for the 11 populations, each with 200 unsexed adults aged 1 to 3 weeks. The insects were observed for 30 min, and the parameters evaluated were the number of insects that succeeded in flying (takeoff) and the flight height. The tests were performed in a room with artificial lighting and at a temperature of 27 °C ± 2 °C for 8 to 17 h. Only acetone was used in the control group.

### 4.10. Statistical Analysis

The concentration–mortality data were submitted to probit analysis (PROC, PROBIT; SAS Institute, 9.0, Minato, Japan). The confidence intervals for toxicity ratios (TRs) were calculated according to Robertson et al. [54], and the values of lethal concentrations were considered significantly different if their 95% confidence interval did not reach the value 1.

The respiratory rate (CO_2_ production), r_i_, flight height, and takeoff data for PHEO-treated and control populations were subjected to variance analysis (PROC GLM; SAS Institute, 9.0). Subsequently, the Scott–Knott test for means was used to cluster the results among the populations (*p* < 0.05) [55], and the F-test was applied to compare the treatments with and without PHEO (*p* < 0.05) (PROC GLM; SAS Institute, 9.0). The body mass data were subjected to analysis of variance (PROC GLM; SAS Institute, 9.0) and grouped using the Scott–Knott test for means [55].

The results of the walking variables (distance, time, speed, and walking time proportion) were subjected to analysis of variance (PROC GLM; SAS Institute, 9.0). The Scott–Knott test was used to determine behavioral variations among the populations (*p* < 0.05), and the F-test was employed to determine behavioral variations between the PHEO-treated and partially PHEO-treated arenas (*p* < 0.05).

## Figures and Tables

**Table 1:** Chemical composition and relative concentrations of the compounds identified in PHEO using gas chromatography coupled with mass spectrometry analysis (GC-MS).

Compounds	RI ^a^ (Literature)	RI ^b^ (Calculated)	Relative %
p-Cymen-8-ol	1179	1184	1.20
Safrole	1285	1292	93.00
(E)-Caryophyllene	1417	1415	0.69
Bicyclogermacrene	1500	1493	2.05
Pentadecane	1500	1498	1.60
Spathulenol	1577	1573	1.46

^a^ Relative retention index according to previous research [31] and the NIST-14 Library. ^b^ Retention index experimentally determined using a homologous series of C_7_–C_30_ alkanes (Kovats index).

**Table 2:** Relative residual toxicity of PHEO to Brazilian populations of *Sitophilus zeamais* adult insects. The PHEO concentration ranged from 140.00 to 1000.00 μL kg^−1^ (24 h exposure).

Populations	Slope ± SEM ^1^	LC_50_ (FI 95%) μL kg^−1^	TR (FI 95%) LC_50_	LC_95_ (FI 95%) μL kg^−1^	TR (CI 95%) LC_95_	χ^2^	*p*
Cristalina, GO	3.95 ± 0.32	255.79 (234.56–276.33)	-	667.49 (587.62–789.10)	1.06 (0.88–1.26)	4.69	0.20
Crixás, GO	4.21 ± 0.33	288.88 (267.23–310.20)	1.13 (0.92–1.39)	710.95 (628.85–835.11)	1.13 (0.92–1.38)	3.76	0.29
Picos, PI	4.27 ± 0.34	324.28 (301.70–347.39)	1.27 (1.05–1.54)	787.68 (692.73–933.39)	1.25 (1.01–1.53)	4.14	0.25
Viçosa, MG	4.34 ± 0.34	325.44 (303.00–348.25)	1.27 (1.06–1.53)	778.80 (686.85–918.70)	1.23 (1.02–1.49)	5.85	0.12
Tunápolis, SC	5.92 ± 0.45	333.47 (314.42–352.32)	1.30 (1.09–1.56)	632.45 (578.57–709.31)	-	2.81	0.42
Uirapuru, GO	4.20 ± 0.35	336.36 (312.81–360.53)	1.31 (1.10–1.58)	828.06 (724.65–989.55)	1.31 (1.06–1.63)	3.90	0.27
Recife, PE	4.52 ± 0.36	337.76 (315.46–360.76)	1.32 (1.10–1.59)	781.31 (691.01–918.66)	1.24 (1.01–1.52)	4.08	0.25
Machado, MG	5.16 ± 0.40	352.60 (331.46–374.31)	1.38 (1.16–1.64)	734.71 (659.90–845.54)	1.16 (0.96–1.40)	5.67	0.13
Londrina, PR	4.89 ± 0.40	383.20 (359.92–407.99)	1.50 (1.25–1.79)	832.01 (736.24–980.09)	1.32 (1.06–1.63)	4.85	0.18
Jacarezinho, PR	3.60 ± 0.28	421.43 (387.34–459.56)	1.65 (1.38–1.97)	1206.00 (1023.00–1497.00)	1.91 (1.54–2.36)	4.12	0.25
Juiz de Fora, MG	6.85 ± 0.53	487.75 (465.72–510.67)	1.91 (1.62–2.25)	847.77 (780.09–94.90)	1.34 (1.12–1.60)	4.15	0.25

^1^ Standard Error of the Mean. LC = Letal concentration. TR = Toxicity ratio. FI = Fiducial interval.

## Data Availability

The datasets used and/or analyzed in this study are available upon request addressed to the corresponding author.
